# Impact of ChAdOx1 nCoV-19 (Covishield™) Vaccination: How Long Will It Persist?

**DOI:** 10.1155/2022/4729844

**Published:** 2022-05-31

**Authors:** Leimapokpam Sumitra Devi, Moumita Sardar, Mukesh Sharma, Manisha Khandait

**Affiliations:** Department of Microbiology, Faculty of Medicine and Health Sciences, SGT University, Gurugram, Haryana, India

## Abstract

An increase in COVID-19 immunization coverage has been linked to a decrease in the average case fatality rate. As a result, further research is needed to determine the persistence and duration of vaccine-induced protective antibodies in order to assess the effectiveness of COVID-19 vaccinations. The present study aimed to determine the COVID-19 IgG antibodies among healthcare workers (HCWs) before and after the ChAdOx1 nCoV-19 (Covishield™) vaccination. A total of 150 HCWs who had received the Covishield™ vaccine were assessed after obtaining written informed consent. Blood samples were drawn at three time points, namely, within one week prior to first dose of vaccination, prior to second dose of vaccination (28–33 days after the first dose of vaccination), and 90–95 days after the second dose of vaccination for detecting neutralizing antibodies, i.e., IgG antibodies by ELISA. The overall baseline seropositivity among the HCWs was found to be 28% (*n* = 42), assessed by the sample collected prior to first dose of COVID-19 vaccination. The seroconversion rate was reported to be 80% (*n* *=* 120) one month after the first dosage and increased to 92.7% (*n* *=* 139) three months later. Additionally, there was a significant gradual increase in the IgG concentrations postvaccination in majority of the study participants. In those HCWs who had prior history of SARS-CoV-2 infection, significantly higher antibody level was observed compared to antibody-naive individuals. Fever, pain or swelling at the site of injection, and headache were the most frequently reported adverse events following vaccination among the study participants. Regardless of prior SARS-CoV-2 positivity, two doses of the Covishield^TM^ vaccine elicited a protective neutralizing antibody response that lasted for three months after the second dose of vaccination.

## 1. Introduction

Severe acute respiratory syndrome coronavirus-2 (SARS-CoV-2), which is responsible for causing coronavirus disease 2019 (COVID-19), first emerged in Wuhan, China, in December 2019 and has spread to every continent of the world, proving itself as one of the greatest pandemics in human history. The current pandemic has affected 266,504,411 people with 5,268,849 deaths globally as of 8 December 2021 [1]. The USA has been the most widely affected region, with 48,982,584 confirmed cases and 783,433 deaths, followed by India, reporting 34,656,822 cases with 473,952 deaths, and Brazil, reporting 22,147,476 confirmed cases with 615,744 deaths [1]. The newly discovered infectious disease is often associated with extensive morbidity and mortality; thus, to curb this health crisis, a safe and effective vaccine is the crucial tool. Large-scale research on vaccine development programme became the need of the hour globally, and along with various partners, the WHO worked vigorously to develop, manufacture, and deploy safe and effective COVID-19 vaccines [2].

Coronavirus spike protein (S protein) is a major target for COVID-19 vaccines, particularly its receptor-binding domain (RBD), as SARS-CoV-2 neutralizing antibodies binding to this domain prevent the conformational change required by the virus to efficiently bind ACE2 and enter human cells, thus inhibiting virus attachment [3, 4]. The majority of the current vaccine candidates are being evaluated for their potential to induce humoral immunity (measured by the magnitude of binding antibodies to the coronavirus spike protein) and the quality of the antibody responses in terms of neutralizing abilities. Currently, there are 22 COVID-19 vaccine candidates that are undergoing assessment for WHO Emergency Use Listing (EUL)/prequalification (PQ) evaluation process [2]. By the end of year 2020, several vaccine candidates have reached phase 3 efficiency and received green signal for emergency use authorization (EUA) [5–7]. Since December 2020, widespread COVID-19 vaccination programmes with a focus primarily on high-risk groups such as healthcare workers (HCWs) and other frontline workers, elderly, or those with comorbidities have achieved a milestone of 3,984,596,440 individuals being vaccinated globally as of 6 August 2021 [2]. At least one vaccine dose has been received by 30% of the world population, while 15.5% have been fully vaccinated [8]. In India, the first phase of administration of COVID-19 vaccine began on 16th January 2021; since then, a total of 50,86,64,759 vaccine doses have been administered in various phases, of which 8.2% (*n* = 11,29,97,166) of the population have received both the doses (fully vaccinated) [9]. Initially, India approved AZD1222-ChAdOx1-S (Covishield) (manufactured under licence from the Oxford–AstraZeneca by Serum Institute of India) and BBV152 Covaxin (manufactured by Bharat Biotech, India, developed in collaboration with the Indian Council of Medical Research). Later, in April 2021, Gam-COVID-Vac/Sputnik V developed by Gamaleya National Research Institute of Epidemiology and Microbiology, Moscow, was approved for vaccination in India [10]. As of August 7, 2021, 1,031,659 healthcare workers had received their first dose of vaccine, with 79,72,650 fully vaccinated [9].

Globally, mass vaccination has been advocated as a critical component to curb the pandemic; however, vaccination alone has its limitations. Though COVID-19 vaccination provides a high degree of protection against disease severity, hospitalization, and death, it does not provide 100% protection as breakthrough infections may occur even after being fully vaccinated [11]. Additionally, with the emergence of COVID-19 virus variants which are likely to be more dangerous and transmissible, the dilemma persists regarding the effectiveness of approved vaccines or those currently in the developmental stage. Fortunately, because of the broad immune response that vaccines induced, it is unlikely to make vaccines completely ineffective [11]. Association of an increase of 10% COVID-19 vaccine coverage with a reduction of the average case fatality rate by 7.6% has been reported by researchers from Taiwan [12]. The longevity or persistence of protective antibodies produced after COVID-19 infection has been reported to be up to 6 months and beyond [13, 14]. Thus, more research is needed to determine the presence and persistence of antibodies developed after COVID-19 vaccination and their protective role, which will finally determine the effectiveness of vaccination. The present study aimed to determine the IgG antibody titers/levels/concentrations (binding antispike antibody) to SARS-CoV-2 among HCWs before and after vaccination. Furthermore, the adverse events following vaccination reported by the study participants have also been assessed.

## 2. Materials and Methods

### 2.1. Study Design and Study Population

The present institutional-based ongoing prospective cohort study was conducted between January 2021 and July 2021 in SGT Medical College Hospital and Research Institute (a tertiary care hospital) in Gurugram district of Haryana, India. Ethical approval for the study was obtained from the Institute Ethics Committee of SGT University, Haryana (SEC/FMHS/F/06/02/21/82). Over a period of seven months (January to July 2021), a total of 173 healthcare workers who had received ChAdOx1 nCoV-19 (Covishield™) manufactured by the Serum Institute of India within the first three weeks after the introduction of COVID-19 vaccination programme in our country were enrolled in the study for evaluation of IgG antibody response against SARS-CoV-2 after obtaining written informed consent. Of the study participants, follow-up samples (as per the study protocol) could be obtained from 150 HCWs, while 23 HCWs who were lost to follow-up were excluded from the study. In addition, those HCWs vaccinated with Covaxin were also excluded from the study. Thus, the demographic, epidemiological, and clinical data were analyzed only for those who could give all the samples at different time points (*n* = 150 HCWs).

### 2.2. Data Collection

Demographic and clinical data, vaccination details, and adverse events following vaccination data were collected from all the study participants. All the information collected from the study participants during the study period was kept confidential to protect the identity of the subjects; thus, analysis was done after deidentifying the data.

### 2.3. Laboratory Analysis

Approximately 3 ml of blood sample was collected for quantitative measurement of anti-COVID-19 IgG antibody at three sampling points, i.e., within one week prior to the first dose of vaccination to determine baseline antibody response (first sampling point), prior to the second dose of vaccination, i.e., 28–33 days after the first dose of vaccination (second sampling point), and 3 months after the second dose of vaccination, i.e., approximately 90–95 days after the second dose of vaccination (third sampling point) to determine the IgG antibody response and persistence. As per the government guidelines for COVID-19 vaccination protocol, during the study period, two doses of vaccines were administered at an interval of 28 days which has been considered as the second sampling point [15].

The SARS-CoV-2 IgG antibodies produced against the spike protein (S1-RBD protein) were assayed using the ELISAFE *Q* COVID-19 IgG quantitative ELISA detection kit as per the manufacturer's instructions. The specified performance characteristics (100% sensitivity and the specificity) of the assay are mean of ICMR testing lab, other external government labs, and internal testing experiments as per the manufacturer [16]. The cutoff value was calculated using the formula “cutoff calculations = 0.2 + average OD value of negative control,” while the antibody index (AI) was determined using the formula “AI = observed sample OD at 450 nm/cutoff.” Thus, the results were calculated as AI and interpreted as per the interpretation criteria, i.e., AI <0.9 was interpreted as negative, 0.9–1.1 as equivocal, and >1.1 as positive. A standard curve was plotted from 8 dilutions of IgG standard (provided with the kit) against S1-RBD of SARS-CoV-2 considering all dilutions. Antibody concentrations were calculated considering the dilution factor and expressed as arbitrary units (AU/ml) and interpreted the results. Antibody levels of >110 AU/ml were considered seropositive, while antibody levels of <90 AU/ml were considered seronegative [16].

### 2.4. Statistical Analysis

Data were entered into Microsoft Excel 2010 and analyzed using Stata version 12.0. Seroprevalence was reported as percentage. Categorical variables were compared by univariate analysis using the *χ*^2^ test, while continuous variables were expressed as mean ± standard deviation, median, and interquartile range (IQR) and compared by Student's *t*-test. A *P* value of < 0.05 was considered statistically significant.

## 3. Result

### 3.1. Demographic and Clinical Data

A total of 150 healthcare workers including doctors, nurses, laboratory technicians, and office assistants working in SGT Hospital who had received ChAdOx1 nCoV-19 (Covishield™) were included in the study. The age group of the participants ranged from 21 to 73 years (mean age, 37.1 ± 13.8 years), with majority of them belonging to the age group of 21–40 years (65.3%). Most of the participants were males, with a male:female ratio of 1.8 : 1 (males, *n* = 97; females, *n* = 53). Among the participants, the majority were doctors (32%, 48/150), followed by nurses (26%, 39/150), laboratory technician (17.3%, 26/150), and office assistants (24.7%, 37/150). A small number of participants had comorbidities, namely, diabetes mellitus (2%, 3/150), hypertension (5.3%, 8/150), and 2% (3/150) HCWs revealed history of cardiac disease and other chronic illnesses. Among the study participants, 38 (25.3%) had prior history of COVID-19 infection themselves, while 42 (28%) revealed COVID-19 infection among their family members (SARS-CoV-2 RT-PCR positive). A few participants (4%) revealed death among the family members due to COVID-19 in the second wave. All the participants had received Covishield™ as it was the available vaccine in the present institute.

### 3.2. Evaluation of SARS-CoV-2 IgG Antibody (Seropositivity in Samples Collected at Different Time Points)

There was a significant gradual increase in the seropositivity between the three sampling points, i.e., prior to the 1^st^ dose, prior to the 2^nd^ dose, and 3 months after the 2^nd^ dose (*P* < 0.05). The baseline seropositivity among the HCWs was found to be 28% (*n* = 42), assessed by the sample collected prior to the first dose of COVID-19 vaccination. The antibody index observed ranged between 1.1 and 14.3 during the first sampling point; among them, 59.5% (25/42), 19% (8/42), and 21.5% (9/42) showed AI between 1.1 and 5 (2.2 ± 0.8), 5.1 and 10 (6.9 ± 1.3), and 10.1 and 16 (9.4 ± 3.6), respectively (Table 1). The seropositivity was significantly increased to 80% (*n* = 120) when compared with the first sampling point (*P* < 0.05). Additionally, the antibody concentration (AI) was also higher in the second sample compared to the first sample ranging between 1.1 and 15.8; among them, 43.3% (52/120), 37.5% (45/120), and 19.2% (23/120) showed AI between 1.1 and 5 (2.9 ± 0.9), 5.1 and 10 (7.1 ± 1.5), and 10.1 to 16 (12.4 ± 3.3), respectively. On assessment of IgG antibody response and persistence of the antibody 90–95 days after the second dose, a high degree of seropositivity was noted (*n* = 139, 92.7%) with a higher concentration of antibodies, while there were eleven individuals who had antibody index <0.9 (seronegative). The antibody index ranged between 1.7 and 16; among them, 25.2% (35/139), 49.6% (69/139), and 25.2% (35/139) showed AI between 1.7 and 5 (3.3 ± 1.1), 5.1 and 10 (8.1 ± 2), and 10.1 and 16 (12.8 ± 1.8), respectively. Corresponding calculated AU/ml values for median titer level for samples collected at three different sampling points increased significantly after the second dose, from a median of 412 AU/mL (IQR: 1503.8–226.4) to 1129.9 AU/mL (IQR: 5902.6–411.3) and 2823.2 AU/ml (IQR: 14611.8–820.4) (*P* < .001).

When compared to the baseline antibody level, the antibody level in the sample obtained before the second dose of vaccination was shown to be waning in four cases (2.7%). Furthermore, the antibody level was higher in six individuals in samples taken before the second dose of vaccination; however, it decreased in the third sample, which was taken 90–95 days following the second dosage. Two HCWs were found to be seronegative in all the samples collected at different time points.

### 3.3. Comparison of SARS-CoV-2 IgG Antibody among Participants with Prior History of COVID-19

Out of the 42 HCWs with prior history of COVID-19, 34 (81%) were symptomatic and 8 (19%) were asymptomatic (data not shown in the table).  Table 2 shows the time frame for those who tested positive for COVID-19, majority of them had COVID-19 infection in September and October 2020. There was increase in the IgG antibody index over time, highest being detected in the sample collected three months after the second dose of vaccine (Table 2).

### 3.4. Assessment of SARS-CoV-2 Seropositivity among HCWs Based on Exposure to COVID-19 Patients or COVID-19 Samples

Baseline seropositivity among the different categories of HCWs was assessed considering IgG antibody positivity detected in samples collected prior to the first dose of the COVID-19 vaccine. The subdivision of different categories of HCWs based on their degree of occupational exposure to COVID-19 patients or samples collected for diagnosis has been depicted in Table 3. Out of the 42 participants with baseline seropositivity, the majority of them (54.5%) were laboratory technicians who handled COVID-19 sample in diagnostic laboratory or posted in COVID-19 sample collection area. All the nurses included in the study had been engaged in caring of COVID-19 patients; of these, 15 (50%) were seropositive, while among the doctors who had exposure to COVID-19 patients, 33.3% (*n* = 10) were found to be SARS-CoV-2 seropositive. There were significant differences in the baseline seropositivity among the exposed and nonexposed subgroups of doctors and laboratory technicians (*P* < 0.05). Seven office assistants from the exposed group were found to be baseline seropositive, but none from the nonexposed group were baseline seropositive.

### 3.5. COVID-19 Vaccine Breakthrough Infection and Adverse Events after the COVID-19 Vaccination

A small proportion of participants (3.3%, *n* = 5) had vaccine breakthrough infection even after receiving two doses of COVID-19 vaccine, with mild symptoms of the infections; thus, none of them required hospitalization.

**Figure 1:**
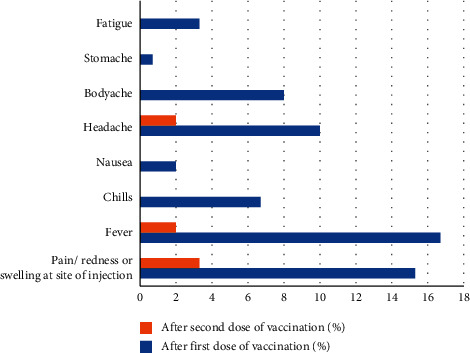
Adverse events reported following COVID-19 vaccination.

Out of the 150 individuals, 43 (28.7%) experienced mild-to-moderate adverse events after receiving the first dose of COVID-19 vaccine, while 8 (5.3%) revealed mild adverse events after receiving the second dose of vaccine. Following the first dose of vaccine, most common adverse events (mild to moderate) reported among the participants were fever (*n* = 25, 16.7%) followed by pain/redness/swelling at the site of injection (*n* = 23, 15.3%), headache (*n* = 15, 10%), body ache (*n* = 12, 8%), and chills (*n* = 10, 6.7%), while less common adverse events were fatigue (*n* = 5, 3.3%) and nausea (*n* = 3, 2%), and one of the participants experienced stomachache. In contrast to the first dose of vaccine, very few participants had complaints of fever, pain/redness/swelling at the site of injection, and vomiting (Figure 1).

## 4. Discussion

The novel coronavirus responsible for the current pandemic has affected every section of individuals and is often associated with extensive morbidity and mortality globally. Thus, the mankind was looking for a safe and effective vaccine in order to bring the current health crisis to an end. Various types of COVID-19 vaccines were swiftly developed, namely, mRNA encoding SARS-CoV-2 spike protein, viral vector-based, virus-like particle, inactivated virus, and recombinant protein vaccines [17]. Most of the SARS-CoV-2 vaccines may potentially have disease-preventing or disease-attenuating effects but not necessarily sterilizing effects [18]. Mass vaccination was started worldwide with the hope of substantial reduction in mortality and morbidity from COVID-19 and bringing in gradual end to the pandemic. Studies on COVID-19 mass vaccination in Israel and Scotland reported vaccine effectivity with a significant decrease in number of deaths, a decrease in disease severity, and reduced hospitalization rate in those who had received the COVID-19 vaccine [19, 20]. In India, the first phase of COVID-19 vaccination was started on 16 January 2021, with the two vaccines, namely, Covishield™ and Covaxin, targeting three crore HCWs and other frontline workers. The two vaccines were approved by the National Regulatory Body for Emergency Use since January 2021, and later Sputnik V vaccine was approved in April 2021 [9, 10]. In India, the choice of vaccination obtained is based on personal preference and/or availability of the vaccines at the vaccination centers.

In the present ongoing postvaccination follow-up study, all the HCWs vaccinated with Covishield™ were assessed for vaccine-induced IgG antibody response against SARS-CoV-2 spike protein at three sampling points, i.e., within one week prior to the first dose of vaccination, 28–33 days after the first dose of vaccination, and 90–95 days after the second dose of vaccination. The observation of the study showed that two doses of Covishield™ vaccination induce significant antispike IgG antibody among all but 11 participants over a period of time and it still persisted luxuriously in the serum even after three months of the second dose of vaccination irrespective of their age and gender (92.7% seropositivity). A study from Western India evaluated the antispike IgG antibody response after the first dose of Covishield™ and Covaxin among HCWs. The study highlighted that both the vaccines induced immune responses; however, the rates of seropositivity to antispike IgG were significantly higher in Covishield™ vaccinees, highlighting 79.3% seropositivity after the first dose and 95% seropositivity to antispike IgG antibody 21–36 days after the second dose of vaccination [21]. A similar type of study from southern India reported higher seroconversion rate (97.1%) among healthcare workers after 28 days of immunization with the first dose of Covishield™ [22]. A study from Sri Lanka revealed a high rate of seroconversion after single dose of Covishield™ vaccine among HCWs. However, they concluded that immunogenicity induced after a single dose of vaccine was insufficient to aid protection against emerging SARS-CoV-2 variants. In addition, the study also highlighted the varying seroconversion rate according to age group, i.e., lower seroconversion rates in the older age group (>60 years) compared to younger individuals (<60 years) [23]. Age-wise comparison could not be done due to less sample size of the older age group in our study. A multicentric study from India reported a higher seroprevalence rate of 90.3% in those who had received two doses of Covishield™ [24]. A nationwide study from Hungary reported the estimated effectiveness against five different COVID-19 vaccines, i.e., Pfizer-BioNTech, Moderna, Sputnik V, AstraZeneca, and Sinopharm as 83.3%, 88.7%, 85.7%, 71.5%, and 68.7%, respectively [25].

Only 14 (9.3%) of the study participants reported having associated comorbidities; thus, it was not possible to correlate the association of comorbid conditions with a decrease or increase in antibody response after vaccination. A study from Western India reported that there was no significant difference in the seroconversion with regard to any comorbid condition, except those with hypertension, where the seroconversion rate is low and the antibody level as well [13]. A high seroconversion rate (98%) among Sri Lankan HCWs with comorbid conditions such as diabetes, chronic kidney disease, or hypertension was reported [14].

Participants with history of COVID-19 infection had significantly higher antibody responses in terms of seroconversion and antibody titer compared to those without documented COVID-19 infection (SARS-CoV-2-naive individuals), probably because of vaccination having a booster effect on persisting immunity due to natural infection. Similar observations have been reported in studies from India and abroad [17, 21, 22]. Furthermore, the antibody response becomes even higher in those with history of symptomatic SARS-CoV-2 infection than in people who are asymptomatic and in people who are hospitalized than in those who are handled as outpatients [17, 26–28]. In the present study, smaller proportion of HCWs (4%) reported death among their family members due to COVID-19 in 2021, i.e., second wave of the pandemic, which highlighted the devastating situation, infection rapidly spreading across the country with much higher mortality rate seen in the second wave compared with the first wave of the pandemic.

COVID-19 vaccine breakthrough infections have been reported among a few participants (3.3%) in the present study. The symptoms were milder, and all recovered with simple home isolation without any complications or requiring hospitalization. Similarly, a study from Israel reported low rate (2.6%) of breakthrough infections among HCWs [29]. This study also reported mild or asymptomatic breakthrough infections which were evident in the present study also. A slightly increased rate of breakthrough infections (11–13.3%) was reported from northern Indian hospitals among HCWs who had been vaccinated with both the vaccines (Covishield™ and Covaxin) [30, 31]. In our study, deviating immune responses were observed in a few individuals; seroconversion was absent in two individuals (no prior documented COVID-19 infection) after two doses of Covishield™ vaccine, and in six individuals, antibody levels decreased at 90–95 days after the second dose compared to samples collected at 28–33 days after the first dose. Studies have shown lower seroconversion rates in those with hematologic malignancies or those on specific immunosuppressive drugs [32]. However, in the present study, no known underlying comorbid or immunosuppressive conditions were revealed by the study participants. Further studies are needed to assess other underlying causes for waning or absence of seroconversion postvaccination.

In the present study, mild-to-moderate adverse events after receiving the first or second dose of vaccine have been reported in 28.7% and 5.3% participants, respectively. A similar degree of adverse side effects has been reported among Covishield™ recipient HCWs [21]. A study from India reported that higher proportion of HCWs (78.9%) revealed side effects of vaccination, majority of the symptoms being systemic side effects and injection site related problems [33]. A similar study from Czech Republic reported injection site pain (89.8%), fatigue (62.2%), and headache (45.6%) as the most commonly reported postvaccination adverse events [34].

## 5. Limitations

The present study consisted of a small sample size focused only on healthcare workers and a very short follow-up period postvaccination to concretely conclude the efficacy of vaccine. In addition, cellular immunity after vaccination could not be assessed. Furthermore, the persistence of antibody level after 1^st^ dose of vaccination could not be assessed for longer duration as the second dose was administered after 28 days as per the then existing Indian Government guidelines. Whether the antibody levels elicited postvaccination translates to the duration of protection, will confer protection against the variants of concern, and affect the rate of transmission needs further investigation. In addition, duration of antibody response and minimum titer to be attained for protection against COVID-19 will help address the question of whether booster dose or doses are required as mass vaccination drives throughout the country are costly, laborious, and time-consuming.

## 6. Conclusions

Overall, vaccination with two doses of Covishield™ elicited a higher anti-IgG antibody response and protective immunity, which persisted luxuriously even after three months of full vaccination. Additionally, those HCWs with prior history of COVID-19 positivity had higher antibody titers when compared with those naïve individuals with no prior exposure.

## Figures and Tables

**Table 1:** Detection of SARS-CoV-2 IgG antibodies among healthcare workers at various sampling points.

Antibody index range	Sampling point for SARS-CoV-2 IgG antibody detection						Statistical analysis
	Prior to 1^st^ dose		Prior to 2^nd^ dose		3 months after 2^nd^ dose		
	*N* (%)	Mean ± SD	*N* (%)	Mean ± SD	*N* (%)	Mean ± SD	
1.1–5	25 (59.5)	2.2 ± 0.8	52 (43.3)	2.9 ± 0.9	35 (25.2)	3.3 ± 1.1	<0.001
5.1–10	8 (19)	6.9 ± 1.3	45 (37.5)	7.1 ± 1.5	69 (49.6)	8.1 ± 2	0.02
10.1–16	9 (21.5)	9.4 ± 3.6	23 (19.2)	12.4 ± 3.3	35 (25.2)	12.8 ± 1.8	0.004

*P* < 0.05 was considered statistically significant.

**Table 2:** Analysis of COVID-19 seropositivity among HCWs with prior history of COVID-19.

Time period for diagnosis of study participants as COVID-19 positive	Baseline seropositivity among HCWs (*n* = 42) *N* (%)	Mean IgG antibody index in three sampling points Prior to 1st dose	Prior to 2nd dose	3 months after 2nd dose	Statistical comparisons
July-August 2020	7 (16.7)^*∗*^	3.6 ± 1.4	6.8 ± 0.9	7.5 ± 2.4	<0.001
September-October 2020	19 (45.2)^*∗*^	4.2 ± 1.8	7.3 ± 2.5	9.2 ± 2.7	<0.001
November-December 2020	16 (38.1)^*∗*^	7.1 ± 2.6	8.9 ± 2.6	10.4 ± 3.2	0.003

^*∗*^Calculated out of the total number of participants with prior history of COVID-19.

**Table 3:** Baseline SARS-CoV-2 seropositivity among HCWs based on their exposure to COVID-19 patients or samples.

Occupation of the participants	Exposure to COVID-19 patients/samples, *n* (%)			
	Exposed		Nonexposed	
	Total	BLS^*∗*^	Total	BLS^*∗*^
Doctor (*n* = 48)	30 (62.5)	10 (33.3)^1,2^	18 (37.5)	2 (11.1)^1^
Nurse (*n* = 39)	39 (100)	15 (50)^1^	0 (0)	0 (0)
Laboratory technician (*n* = 26)	11 (42.3)	6 (54.5)^1,2^	15 (57.7)	2 (13.3)^1^
Office assistants (*n* = 37)	34 (91.9)	7 (20.6)^1^	3 (8.1)	0 (0)

^*∗*^BLS = baseline seropositive; 1 = calculated out of the total of exposed or nonexposed HCWs; 2 = *P* < 0.05 in exposed vs. nonexposed HCWs.

## Data Availability

All the data generated or analyzed during the present study are included in the research article.
